# Insomnia in Forensic Detainees: Is Salience Network the Common Pathway for Sleep, Neuropsychiatric, and Neurodegenerative Disorders?

**DOI:** 10.3390/jcm13061691

**Published:** 2024-03-15

**Authors:** Adonis Sfera, Kyle A. Thomas, Isaac A. Ogunjale, Nyla Jafri, Peter G. Bota

**Affiliations:** 1 Department of Psychiatry, Patton State Hospital, University of California, Riverside, CA 92521, USA; 2 School of Medicine, California University of Science and Medicine, Colton, CA 92324, USA

**Keywords:** Von Economo neuron, interoceptive awareness, frontotemporal dementia behavioral variant, phenazines

## Abstract

**Highlights:**

**What are the main findings?**

- SN dysfunction is the common denominator of insomnia, schizophrenia (SCZ), and frontotemporal dementia behavioral variant (bvFTD).

**What is the implication of the main findings?**

- The diagnosis of bvFTD is often missed or misdiagnosed in forensic institutions.
- To ensure adequate placement and treatment planning, courts and clinicians require education to differentiate bvFTD from SCZ.

**Abstract:**

Forensic hospitals throughout the country house individuals with severe mental illness and history of criminal violations. Insomnia affects 67.4% of hospitalized patients with chronic neuropsychiatric disorders, indicating that these conditions may hijack human somnogenic pathways. Conversely, somnolence is a common adverse effect of many antipsychotic drugs, further highlighting a common etiopathogenesis. Since the brain salience network is likely the common denominator for insomnia, neuropsychiatric and neurodegenerative disorders, here, we focus on the pathology of this neuronal assembly and its likely driver, the dysfunctional neuronal and mitochondrial membrane. We also discuss potential treatment strategies ranging from membrane lipid replacement to mitochondrial transplantation. The aims of this review are threefold: 1. Examining the causes of insomnia in forensic detainees with severe mental illness, as well as its role in predisposing them to neurodegenerative disorders. 2. Educating State hospital and prison clinicians on frontotemporal dementia behavioral variant, a condition increasingly diagnosed in older first offenders which is often missed due to the absence of memory impairment. 3. Introducing clinicians to natural compounds that are potentially beneficial for insomnia and severe mental illness.

## 1. Introduction

One of the most common sleep disorders in the United States, primary insomnia, is usually defined as long sleep latency, difficulty staying asleep, prolonged nighttime wakefulness, and/or early morning awakening [1]. In prison, approximately 60% of inmates experience insomnia, a prevalence 6–10 times higher than in the population at large [2]. Moreover, insomnia is present in 67.4% of hospitalized patients with severe mental illness, suggesting that the pathways of sleep and neuropathology are highly intertwined [3].

Forensic psychiatric hospitals admit patients with schizophrenia (SCZ) or schizophrenia-like disorders (SLDs) and criminal violations. Insomnia is common in this population and failure to address this condition may increase healthcare expenditure due to medical complications, including metabolic, cardiovascular, and neurodegenerative disorders.

The salience network (SN), comprised of the anterior insular cortex (AIC), anterior cingulate cortex (ACC) and several subcortical nodes, has recently been implicated in the etiopathogenesis of insomnia, SCZ, and neurodegenerative disorders [4,5,6,7,8,9]. SN is comprised of Von Economo neurons (VENs), a special class of large, spindle-shaped cells found only in humans and superior mammals that are believed to drive empathy, social awareness, and emotional intelligence [10].

At the molecular level, incarceration, insomnia, and severe mental illness have been associated with premature cellular senescence, a phenotype marked by increased intracellular iron and mitochondrial damage [11,12,13,14,15,16,17,18]. Premature cellular senescence is driven by the aryl hydrocarbon receptor (AhR), expressed in neuronal cytosol and mitochondria [19,20,21]. Senescent cells upregulate intracellular iron which, in the proximity of cytosolic fats, increases the risk of lipid peroxidation and neuronal demise by ferroptosis [22,23,24]. Ferroptosis is a programmed cell death induced by iron in the context of antioxidant failure marked by the depletion of glutathione peroxidase-4 (GPX-4) [25,26]. GPX-4 is a mitochondrial enzyme which averts ferroptosis by repairing the oxidized phospholipids and cholesterol in mitochondrial and neuronal membranes [27]. Ferroptosis causes mitochondrial swelling, loss of cristae, dissipation of the membrane potential, as well as an increase in membrane permeability, changes that ultimately lead to mitochondrial loss [28]. Mitochondrial dysfunction and loss drive cellular senescence, a phenotype found in insomnia, severe mental illness and frontotemporal lobar degeneration (FTLD) [29,30,31,32]. In addition, insomnia, SCZ, and frontotemporal dementia (FTD) have been connected to impaired phagocytosis of senescent cells by natural killer cells (NKCs) [33,34,35]. Accumulation of senescent cells due to accelerated aging and impaired removal leads to inflammation, a pathology encountered in sleep deprivation, severe mental illness and FTD [36,37,38]. Since mitochondria is a key driver of inflammation, dysfunction or loss of these organelles likely account for these pathologies [39,40].

To compensate for dysfunctional mitochondria, neurons import these organelles from glial cells, especially the astrocyte [41,42]. In large cells, such as VENs, mitochondria are more vulnerable to damage and autophagic elimination as they undergo more wear and tear during their journey through the long axons of these neurons [42]. Due to their small number (around 193,000) and their large sizes, VENs are more susceptible to plasma membrane oxidative stress, which may trigger significant pathology even after a limited neuronal loss, a pathology encountered in frontotemporal dementia behavioral variant (bvFTD) [43].

Since mitochondria are crucial for neuronal function, preserving the integrity of these organelles via membrane lipid replacement (MLR) and other natural strategies is of utmost importance. Microbial phenazines and the novel antioxidant phenothiazine derivatives offer new opportunities to combat insomnia, psychosis, and neurodegeneration at the level of cell and mitochondrial membranes.

### 1.1. Salienve Network in Sleep and Neuropathology

The SN is comprised of ACC and AIC which, along with subcortical nodes in the hypothalamus, thalamus, striatum, and midbrain, process salient stimuli [44,45]. SN functions as a switch between exteroception and interoception or central executive network (CEN) and default mode network (DMN), depending on stimulus relevance [46]. Switching from CEN to DMN and vice versa is impaired in severe mental illness, insomnia, and neurodegenerative disorders [47]. Several antipsychotic drugs are known to lower the salience assignment to objects and events, likely restoring SN function, which, in turn, may ameliorate insomnia and psychosis [48].

The SN harbors VENs, which are large, corkscrew neurons located in layer V of the AIC and ACC. These non-telencephalic cells are believed to drive prosocial cognition, empathy, and emotional intelligence. As parts of the SN, VENs respond to endogenous or exogenous stimuli in the order of priority. VENs are selectively eliminated in bvFTD, a disorder marked by criminal violations, lack of empathy, poor insight, and sleep impairment [49,50,51,52,53]. In forensic institutions, bvFTD is increasingly diagnosed in older first offenders with no previous criminal history and often coexists with insomnia and altered eating habits.

Under physiological circumstances, sleep is driven by the ventrolateral preoptic nucleus (VLPO) of the anterior hypothalamus which releases inhibitory neurotransmitters, including γ-aminobutyric acid (GABA), and galanin [54]. The opposing system, orexin (hypocretin) neurons in the lateral hypothalamus, inhibits VLPO [55,56,57]. In addition, orexin/hypocretin neurons induce wakefulness by blocking melanin concentrating hormone (MCH), a somnogen released by the hypothalamus and zona incerta [58,59]. Orexin and DA, the key players of saliency, have been implicated in the neuropsychiatric disorders associated with sleep disturbances, including narcolepsy, attention-deficit/hyperactivity disorder (ADHD), and Parkinson’s disease (PD) [60]. Histamine is another wakefulness-promoting neurotransmitter implicated in SCZ and a novel target for treating negative and cognitive symptoms [61].

To better comprehend the pathogenesis of insomnia, it is necessary to study the pathways of wakefulness, a brain state driving self-awareness and probably consciousness [62]. Early studies on this subject have focused on the locus coeruleus, midbrain tegmentum, pons, and parabrachial nucleus, as neurons in these regions are active during wakefulness [63,64]. In the early 1900s, while studying encephalitis lethargica, Constantin von Economo found that lesions in the posterior hypothalamus were associated with sleep, hypothesizing that this area contained the “center of wakefulness” [65,66,67].

Fatal familial insomnia (FFI), a rare autosomal dominant disease, is marked by hypometabolism and neuronal loss in the thalamus and ACC, linking this condition to the SN [68,69,70,71,72]. The role of SN in sleep physiology and pathology is further highlighted by the anesthetics, especially propofol, which lower salience processing, inducing sleep [68,69,70,71,72,73,74,75,76,77,78]. Moreover, recent studies on sleep-deprived human volunteers and patients with primary insomnia demonstrated altered connectivity in AIC, further linking SN to sleep and wakefulness [79,80]. Furthermore, several preclinical studies are in line with the findings in humans, implicating the SN in slumber homeostasis [74,81].

Aside from insomnia and neuropsychiatric pathology, the SN connectivity is disrupted in neurodegenerative disorders, including Alzheimer’s disease (AD), Parkinson’s disease (PD), and bvFTD, suggesting that insomnia and neuropathology are highly intertwined [82,83,84,85,86]. Indeed, dysfunctional AIC and ACC connectivity may account for the criminal violations in patients with bvFTD, in which breaking the law may often be the initial dementia symptom [87,88].

### 1.2. Salience Network in Frontotemporal Dementia Behavioral Variant

The second most common neurodegenerative disorder after AD, bvFTD, is marked by inappropriate emotional responses and disinhibited behaviors, often leading to criminal violations, as this pathology targets VENs selectively [52,89]. In forensic institutions, individuals with first incarceration after the age of 55 may suffer from bvFTD, an entity difficult to diagnose as the memory may remain intact for longer periods of time. As a result, bvFTD is often missed or misdiagnosed as antisocial personality disorder (APD), SCZ, or even major depressive disorder [90].

Over the past two decades, the number of senior first offenders has grown in parallel with the prevalence of young-onset dementia (YOD, emergence of symptoms before age 65), a subgroup of neurodegenerative disorders, which may include bvFTD [91,92]. Indeed, recent studies have revealed that the prevalence of bvFTD has increased from 15/100,000 in 2013 to 119 per 100,000 in 2021, mirroring the growing number of forensic detainees with this diagnosis [92,93].

Compared to AD, in which 12% of patients exhibit criminal behavior, bvFTD is associated with a crime rate of 54%, suggesting an acquired psychopathy [94]. Frontotemporal lobar degeneration (FTLD), the pathology driving bvFTD, is associated with impulsivity and criminal violations due to the paucity of “honesty cells”, VENs [95]. The latter is likely due to the autophagy of damaged organelles traveling through the long VENs axons. Indeed, lysosomal aggregates, hallmarks of hyperactive autophagy, were demonstrated in the VENs derived from patients with bvFTD and SCZ, suggesting excessive mitophagy [95,96,97]. Depletion of VENs has been associated with a lack of empathy, aggressive behavior, and criminal violations documented in bvFTD and severe mental illness [51,52]. For example, homicide or attempted homicide have been documented in bvFTD, indicating that criminal behavior and murder can sometimes be the earliest manifestation of this disorder [98,99]. Since VENs are only present in large mammals, including humans, great apes, macaques, cetaceans, and elephants, but not in rodents, these cells are difficult to study in vivo [10]. VENs are larger than pyramidal neurons and drive interoceptive awareness, which is the ability to detect and process internal cues such as heartbeat, respiration and the overall visceral state [100,101]. VENs are components of the SN, an attention-shifting large neuronal assembly that can activate or silence CEN to DMN [102,103].

Recent transcriptomic studies found that VENs express monoaminergic proteins, including vesicular monoamine transporter 2 (VMAT2) and adrenergic receptor α-1A (ADRA1A), suggesting involvement in autonomic functions, including the circadian rhythm [104,105,106]. Indeed, impaired monoaminergic signaling has been documented in insomnia, bvFTD, SCZ, and SLDs, implicating VENs in these pathologies [107,108,109,110,111].

### 1.3. Sleep and Glial Cells

Astrocytes, the most numerous brain cells, communicate with each other via calcium waves, attaining synchronization with neurons and supporting slow-wave sleep [112,113]. Moreover, astrocytes release molecules, including adenosine, lactate, glutamate, GABA, and interleukin-1 (IL-1), which may indirectly influence the status of neuronal cells, inducing sleep [114].

**Figure 1:**
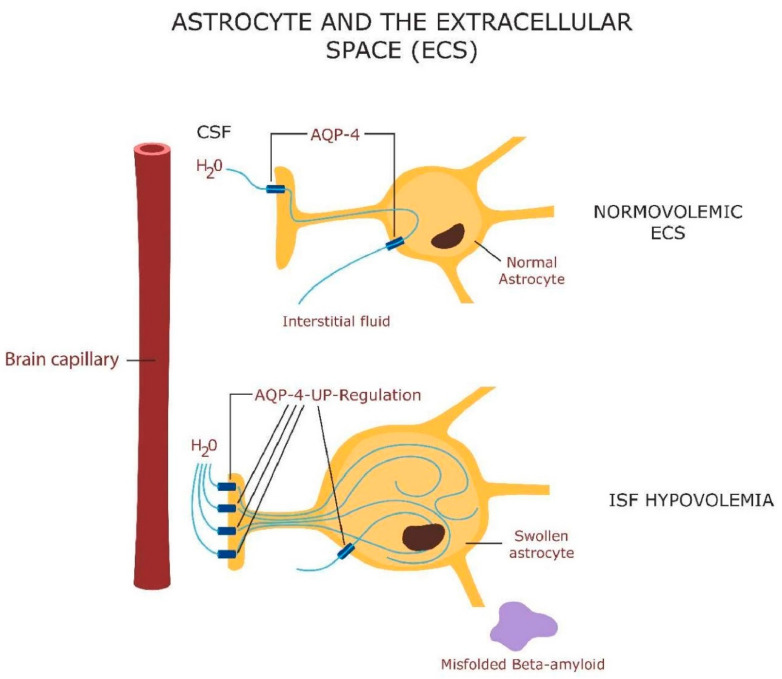
Astrocytes contact cerebral microvessels with their end-feet processes, delineating a pathway for the flow of extracellular fluid, known as the glymphatic system. The volume of interstitial fluid (ISF) in the brain parenchyma varies with the brain work. During high intensity work, AQP-4 water receptors are upregulated in the end-feet, pumping the ISF into astrocytes. This results in low ISF (hypovolemia). During sleep (low-intensity brain work), less ISF enters the astrocyte. The circulation of ISF clears the molecular debris (including beta amyloid) from the extracellular space.

Astrocytes are central to the neurovascular unit (NVU) and bridge the gap between the neuron and brain microvessels, regulating the flow of interstitial fluid through the aquaporin 4 (AQP-4) receptors [115] (Figure 1). The volume of the brain interstitial fluid (ISF) fluctuates in a circadian manner as it flows through the glymphatic system, a mechanism for clearing misfolded proteins during sleep [116]. The glymphatic system can also carry extracellular vesicles containing mitochondria from astrocytes to neurons [117]. Astrocytes support the neurons by generating GPX-4 to avert neuronal death by ferroptosis. GPX-4 functions to repair oxidized lipids and oxysterols, including 7-ketocholesterol (7KCl), toxins that disrupt plasma and mitochondrial membranes, triggering neuronal death [118]. Ferroptosis has been associated with sleep deprivation, indicating that neurons likely import GPX-4 during sleep [119]. As mitochondria play a key role in sleep homeostasis, insomnia may be the result of plasma or mitochondrial membrane oxidation. Indeed, it has been suggested that sleep is necessary for abrogating neuronal oxidative stress [120].

**Figure 2:**
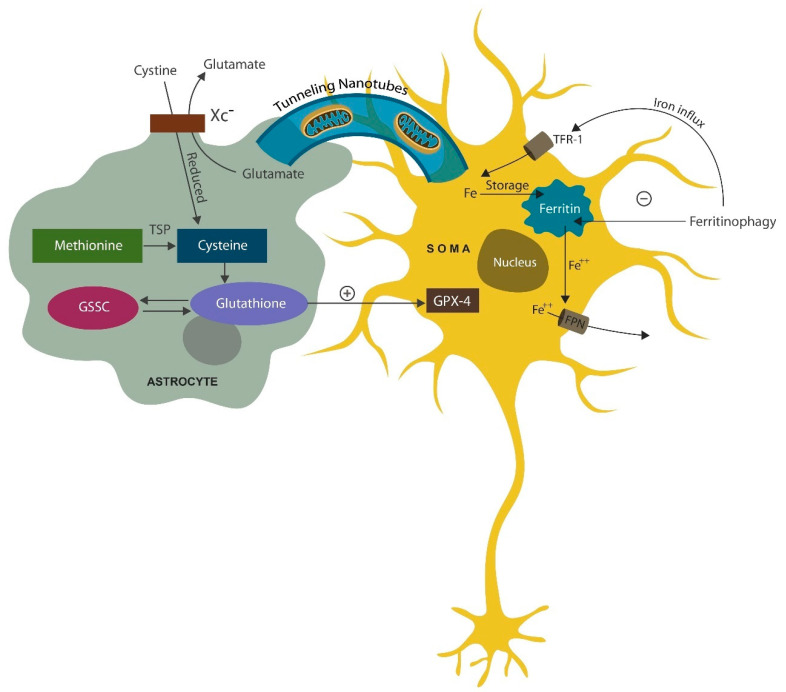
Astrocytes support the postmitotic, long-lived neurons by helping them avert death by ferroptosis and loss of mitochondria. The former is accomplished by exporting GPX-4 to neurons (to repair oxidized lipids), while the latter by exporting healthy mitochondria to neuronal cells (via tunneling nanotubules, extracellular vesicles, or cell–cell fusion). Astrocytes import cystine via cystine/glutamate antiporter (Xc-). Cystine is reduced to cysteine and generates glutathione and GPX-4 (which is transferred to neurons). Cysteine can also be derived from methionine, while glutathione can be generated from cysteine and glutathione disulfide (GSSC). In neurons, iron is stored in ferritin and, when needed, ferritin undergoes ferritinophagy (autophagy) in lysosomes, releasing free iron. Iron ingresses the neuron via transferrin receptor 1 (TRF-1), while the excess intracellular iron is eliminated via ferroportin.

Intracellular iron is stored in ferritin and released for intracellular needs via ferritinophagy (ferritin autophagy) in lysosomes. Several antipsychotic drugs, including haloperidol, accumulate in lysosomes disrupting ferritinophagy, which, in turn, lowers intracellular iron, averting ferroptosis [121,122] (Figure 2). This may highlight a DA-independent, antipsychotic action of haloperidol, suggesting that dopaminergic blockade is not the only psychosis-deterring mechanism of this drug. Indeed, ferroptosis of hippocampal neurons, documented in AD and severe mental illness, is the likely cause of cognitive impairment and negative symptoms in these conditions [123,124]. Prolonged insomnia has been demonstrated to damage the astrocyte which, in turn, may trigger neuronal demise [125]. Moreover, chronic sleep loss was demonstrated to activate both astrocytes and microglia, turning these cells into neurotoxic phenotypes capable of eliminating healthy neurons and synapses [126,127,128].

## 2. Mitochondria and Aryl Hydrocarbon Receptor

Recent studies have implicated mitochondria in the pathophysiology of sleep and neurodegenerative disorders, while the role of these organelles in severe mental illness, including SCZ and SLDs, has been previously established [129,130]. AhR is the master regulator of cellular senescence, a phenotype conducive to aging and neurodegeneration and is expressed by the mitochondrion [19,20,21]. Oxidized lipids in the mitochondrial membrane are AhR ligands, which in conjunction with senescence-upregulated intracellular iron, can trigger ferroptosis and organelle demise [131,132,133,134]. Indeed, lipid peroxides and oxysterols, such as 7KCl, are mitoAhR ligands, contributing to mitochondrial dysfunction and autophagic elimination [135].

AhR is a xenobiotic sensor which regulates cytochrome p450 and binds the environmental toxin, dioxin (2,3,7,8-tetrachlorodibenzo-p-dioxin). Other AhR ligands include somnogens, such as phenazines, melatonin, and tryptophan derivatives, which participate in the physiology of sleep, wakefulness, and the circadian rhythm [136,137,138]. In addition, reactive oxygen species (ROS), known to induce sleep via a redox-sensitive potassium channel, are AhR ligands, bringing this transcription factor in the arena of slumber, mental illness, and neurodegeneration [131,139]. Indeed, microbial phenazines, including pyocyanin and 1-hydroxyphenazine, activate AhR, influencing the transcription of many genes, including those involved in sleep regulation [140,141].

The importance of mitochondria in sleep physiology is further substantiated by the organelle involvement in FFI, as well as in general anesthesia [142,143]. Indeed, general anesthetics are known to inhibit N-methyl-d-aspartate (NMDA) and α-amino-3-hydroxy-5-methyl-4-isoxazolepropionic acid (AMPA) glutamate receptors while stimulating GABA. NMDA and AMPA upregulate intracellular and mitochondrial calcium, inducing cell and organelle demise [144]. Interestingly, elevated mitochondrial calcium, a characteristic of prion diseases, may link these organelles to FFI [145,146]. Indeed, the prion peptide causes calcium inflow via L-type calcium channels, triggering neuronal damage and apoptosis [147]. In contrast, the typical antipsychotic, chlorpromazine, not only induces sleep, but also exerts anti-prion properties, probably by promoting autophagy of the misfolded protein [148,149,150].

Mitochondrial trafficking from astrocytes to neurons supports neuronal bioenergetic needs, especially in large pyramidal cells or VENs. Mitochondria can be imported via cell–cell fusion, tunneling nanotubes (cytoskeletal protrusions reaching to other cells), as well as transported by extracellular vesicles [151,152] (Figure 2). Moreover, astrocytes generate GPX-4 from cysteine obtained via the cystine/glutamate antiporter system (Xc−) or by transmethylation of methionine. Glutathione is generated from cysteine and glutathione disulfide (GSSC) [153] (Figure 2).

Mitochondrial trafficking as well as autophagy (mitophagy) occur during sleep, probably explaining the reason most living beings require rest [154]. Interestingly, serotonin (5-HT) promotes mitochondrial transport in hippocampal neurons, suggesting that antidepressant drugs, serotonin reuptake inhibitors (SSRIs), may “exert their action by supplying healthy mitochondria to stressed neurons [155]. This may imply that ROS accumulation during wakefulness may induce slumber to repair oxidized lipids and import mitochondria from glial cells [120,131,139]. In addition, the accumulation of intracellular microtubule-associated protein tau (MAPT) in VENs likely impairs mitochondrial transport, contributing to bvFTD pathogenesis [156].

### 2.1. Mitochondria-Protective Treatments

The key role of mitochondria in sleep disorders, SCZ, SLDs, and neurodegeneration, highlights the importance of mitoprotective approaches to resuscitate, replace, or increase the import of mitochondria from glial cells [157]. For example, treatment with SSRIs during the early stages of dementia may delay the onset of cognitive decline. Along this line, a recent study found that treatment with SSRIs slowed the conversion of mild cognitive impairment to frank dementia, suggesting that prophylactic treatment with these agents may be beneficial [158]. In addition, natural anti-ferroptosis drugs and iron chelators, such as halogenated phenazines, may improve the course of neurodegenerative disorders, suggesting novel therapeutic strategies [159,160].

### 2.2. Membrane Lipid Replacement (MLR)

MLR refers to the oral supplementation with natural cell membrane glycerophospholipids and kaempferol (3,4′,5,7-tetrahydroxyflavone), a natural flavonoid found in tea, broccoli, cabbage, kale, beans, endive, leek, tomato, strawberries, and grapes [161]. Kaempferol is a glycogen synthase kinase-3β (GSK-3β) inhibitor which prevents sleep deprivation-induced cognitive decline [162,163]. Like lithium and several antipsychotic drugs, kaempferol blocks GSK-3β, an enzyme previously implicated in SCZ and circadian rhythm disorders, suggesting that this natural compound may exert antipsychotic properties without the adverse effects of conventional therapeutics [164,165,166,167].

The aim of MLR + kaempferol is the gradual replacement of damaged phospholipids and oxysterols from neuronal and/or mitochondrial membranes with natural glycerophospholipids and a polyphenol. Indeed, oxidized membrane lipids have been implicated in SCZ, SLDs, insomnia, and neurodegeneration, while MLR and kaempferol offer a dual mechanism of action: (1) elimination of lipid peroxides and (2) GSK-3β inhibition [168]. Replacing oxidized plasma and/or mitochondrial membrane fats with healthy natural lipids averts deformation of the neuronal membrane and misalignment of neuroreceptors. Conversely, oxidized membrane lipids and ferroptosis alter the biophysical properties of membranes, disrupting neuronal functions [169].

### 2.3. Phenazines and Phenothiazine Derivatives

Several natural phenazines and phenothiazines are neuroprotective, improve sleep, and delay neurodegenerative processes. For example, geranyl-phenazine is a naural acetylcholinesterase inhibitor which exerts antipsychotic effects via muscarinic receptors. Indeed, a new class of antipsychotic drugs is currently being developed for SCZ and a patent exists for treating sleep disorders by upregulating acetylcholine [170,171,172] (WO2005016327A2). Other natural phenazines with neuroprotective functions include baraphenazines A–G fused compounds derived from Streptomyces sp. PU-10A which likely possess antipsychotic properties [173]. Moreover, several natural phenazines, including baraphenazines, leucanicidin and endophenasides, exert antimicrobial, anticancer activity, and very likely possess antipsychotic properties [173,174,175].

Natural antipsychotic and phytotherapeutic compounds are not only devoid of extrapyramidal adverse effects but more accepted by many patients who often dread or distrust pharmaceuticals.

Synthetic phenazine derivatives consist of over 6000 compounds, exerting antimicrobial, antiparasitic, neuroprotective, anti-inflammatory, and anticancer activities [176,177,178]. To the best of our knowledge, natural or synthetic phenazines have not been tested for SCZ, insomnia, or neurodegeneration. Pontemazines A and B are neuroprotective phenazine derivatives that, in animal studies, have rescued hippocampal neurons from glutamate cytotoxicity, highlighting their pro-cognitive properties which could benefit patients with negative symptoms of SCZ or neurodegenerative disorders [176].

**Figure 3:**
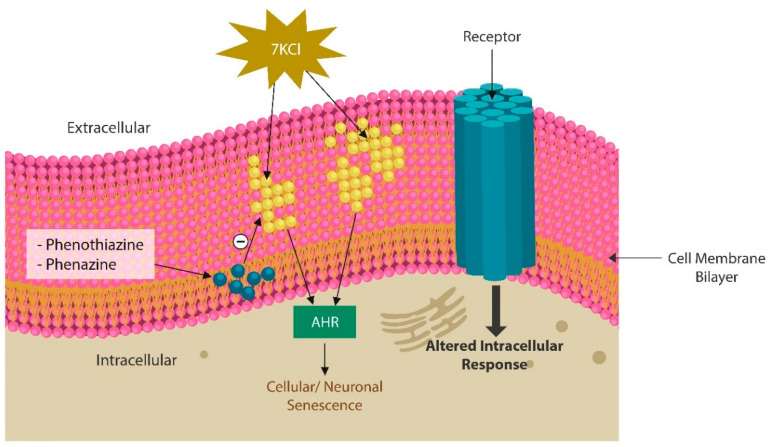
The lipid bilayer of neuronal membrane is easily oxidated when intracellular iron is upregulated. Oxysterols, including 7-Ketocholesterol (a toxic oxide), and oxidated phospholipids alter the biophysical properties of cell membranes, disrupting neurotransmission. In addition, oxidized lipids activate AhR, triggering premature neuronal senescence. Phenazines, phenothiazines, and their derivatives, intercalate themselves into the lipid bilayer, repairing the lipids in cellular and/or mitochondrial membranes.

Synthetic phenazines exert antioxidant and radical-scavenging properties, and inhibit lipid peroxidation, suggesting beneficial effects in severe insomnia, mental illness and neurodegeneration [179,180] (Figure 3). Moreover, halogenated phenazines act as iron chelators, likely preventing neuronal ferroptosis [181]. We believe that pontemazines and halogenated phenazines should be assessed for antipsychotic/anti-neurodegenerative properties.

**Figure 4:**
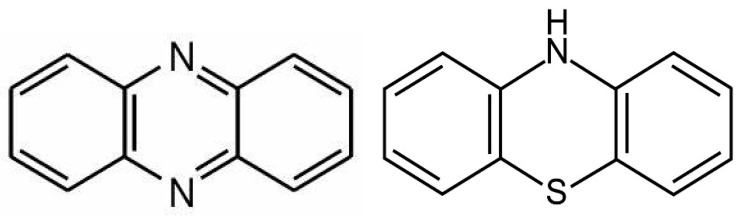
Phenazine vs. phenothiazine: similarities and differences.

From the biochemical standpoint, phenazines are almost identical to phenothiazine antipsychotics and likely possess similar properties (Figure 4). Phenothiazines are typical antipsychotic drugs utilized primarily for SCZ and SLDs, which block dopaminergic transmission at the level of postsynaptic neuron. Several phenothiazines influence other receptors, including adrenergic, histaminergic, and cholinergic, exerting various clinical effects as well as adverse reactions. Aside from psychotic disorders, phenothiazines are also used for the treatment of migraine headaches, hiccups, nausea, vomiting, and cancer [182]. Like phenazines, phenothiazines intercalate themselves into the lipid bilayer of plasma and mitochondrial membranes, disrupting the curvature and receptor alignment on neuronal/mitochondrial surfaces [183] (Figure 3). In contrast, oxidized lipids, including 7-ketocholesterol (7KCl), form looped structures, generating membrane curvatures and pores that may trigger cell death [184].

Antioxidant phenothiazine and their derivatives have recently been developed for cancer, cardiovascular disease (CVD), *Mycobacterium leprae* and other antibiotic-resistant microbes [185,186].

Phenothiazine derivatives exert anti-peroxidation properties and protect against lipid pathology and ferroptosis, suggesting efficacy as antipsychotic drugs [187]. In addition, antioxidant phenothiazines are likely beneficial for insomnia and neurodegenerative disorders, suggesting that these compounds should be tested for neuropsychiatric pathology [186].

Propenylphenothiazine is a potent antioxidant with electron-donor capability that could prevent gray matter loss, a hallmark of SCZ and SLDs [188,189]. Electron-donating psychotropic drugs have been known to preserve the brain volume, suggesting that propenylphenothiazine may treat psychosis without reducing the gray matter volume. The majority of conventional antipsychotic drugs are electron-acceptors which often lower the brain volume as documented by many neuroimaging studies [190]. An even newer category of tetracyclic and pentacyclic phenothiazines with antioxidant properties has recently been developed, suggesting likely efficacy for cognitive impairment and negative SCZ symptoms. Moreover, the N10-carbonyl-substituted phenothiazines were demonstrated to inhibit lipid peroxidation, suggesting superior antipsychotic efficacy [191].

Natural and some synthetic phenazines and novel antioxidant phenothiazines have not been tested for SCZ, insomnia or neurodegenerative disorders but are likely efficient somnogens and antipsychotics. For example, synthetic phenazines, known as pontemazines A and B, rescued hippocampal neurons from glutamate cytotoxicity in rodents, highlighting their pro-cognitive properties which could benefit patients with negative symptoms of SCZ [192].

### 2.4. Natural Antioxidants

SCZ and SLDs have been associated with premature cellular senescence, a phenotype marked by shortened telomeres, accumulation of macromolecular aggregates, increased level of senescence-associated β-galactosidase (SA-β-gal) and a detrimental secretome known as senescence-associated secretory phenotype (SASP).

#### Natural Antioxidant Foods

Antioxidants are major players in repairing damages to macromolecules, opposing pathological events associated with cellular senescence (Table 1). Since AhR is the master regulator of cellular senescence and responds to external pollutants (such as polycyclic aromatic hydrocarbons (PAHs) as well as internal toxins, including oxidized lipids, antioxidants likely have the opposite effect.

### 2.5. Mitochondrial Transfer and Transplantation

Early studies on mitochondrial transplantation from the 1980s utilized co-incubation of various cell types with naked mitochondria, hoping that cells would internalize the organelles from the extracellular environment [202,203,204]. Later, HeLa cells and mesenchymal stem cells were used as mitochondrial sources and found that successful organelle uptake occurred in a short time interval of 1–2 h [205,206,207]. At present, mitochondrial transplantation into cardiomyocytes has been accomplished successfully and confirmed by mitochondrial DNA (mtDNA) detected in host cells [208,209].

Mitochondrial transplantation and neuronal rescue from ferroptosis have been performed successfully in both animals and humans, suggesting a novel strategy for neurometabolic disorders [210]. To our knowledge, mitochondrial transplantation has not been attempted in sleep disorders, while in mental illness, it has been tried in animal models only [132]. Trafficking mitochondria from astrocytes and microglia to neurons can take place spontaneously after brain injuries, reflecting a likely compensatory mechanism to preserve neuronal viability [211]. In addition, it has been established that SSRIs, GJA1-20K, and CD38 signaling can facilitate mitochondrial transfer, emphasizing potential strategies for insomnia, severe mental illness, and neurodegeneration [210,211].

## 3. Conclusions

Forensic detainees with severe mental illness and comorbid insomnia age at an accelerated pace, suggesting that premature cellular senescence, a characteristic of SCZ, may comprise the common pathway where sleep and mental illness intersect. Loss of neurons due to impaired sleep may trigger the premature development of dementia and other age-related conditions, known to occur earlier in life compared to the general population. These comorbidities increase healthcare expenditures and shorten patients’ lifespan; thus, identifying and treating these conditions early is crucial.

YOD, a category of neurodegenerative disorders which include bvFTD, has been on the rise over the past few decades, as evidenced by the increased number of first offenders before the age of 65. Selective loss of VENs in bvFTD is likely due to the large size of these cells, predisposed to peroxidation of plasma membrane lipids and mitochondrial loss by dysfunctional autophagy.

At the molecular level, AhR is the equivalent of VENs, as this protein responds to both endogenous and exogenous ligands, including lipid peroxides and other insomnia and psychosis-related molecules.

Antioxidants and phenazine and phenothiazine derivatives are AhR ligands, highlighting potential natural treatment strategies against psychosis, insomnia, and neurodegeneration.

## Figures and Tables

**Table 1:** SCZ-relevant antioxidants and sources.

Antioxidants	Source	References
Lycopene	Grape skin, guava, grapefruit, blueberries, tomatoes	[193]
Apigenin	Cabbage, blueberries, acai berries	[194]
Phenolic acid	Oilseeds, cereals, grains	[195]
Curcumin	chicken, beef, tofu, vegetables	[196]
Epigallocatechin gallate	Apples, blackberries, broad beans, cherries, black grapes, pears, raspberries, and chocolate	[197]
Berberine	Oregon grape, phellodendron, and tree turmeric.	[198]
Quercetin	Fruits, apples, onions, parsley, sage, tea, and red wine	[199]
Kempferol	Fruits and vegetables.	[200]
Tocopherols	Oilseed, cereals, eggs, deary products	[201]

## Data Availability

Not applicable.
